# Neuropeptide Y inhibits the trigeminovascular pathway through NPY Y_1_ receptor: implications for migraine

**DOI:** 10.1097/j.pain.0000000000000571

**Published:** 2016-03-25

**Authors:** Margarida-Martins Oliveira, Simon Akerman, Isaura Tavares, Peter J. Goadsby

**Affiliations:** a Headache Group, Basic and Clinical Neuroscience, Institute of Psychology, Psychiatry and Neuroscience, King’s College London, London, United Kingdom; b Department of Neurology, University of California, San Francisco, San Francisco, CA, USA; c Department of Experimental Biology, Faculty of Medicine of University of Porto, and Institute of Investigation and Innovation in Health (I3S), University of Porto, Porto, Portugal; S. Akerman is now with the Department of Oral and Maxillofacial Pathology, Radiology and Medicine, New York University College of Dentistry, New York, NY, USA

**Keywords:** Neuropeptide Y, Migraine, Trigeminovascular, Headache, Appetite

## Abstract

Migraine is a painful neurologic disorder with premonitory symptomatology that can include disturbed appetite. Migraine pathophysiology involves abnormal activation of trigeminocervical complex (TCC) neurons. Neuropeptide Y (NPY) is synthesized in the brain and is involved in pain modulation. NPY receptors are present in trigeminal ganglia and trigeminal nucleus caudalis suggesting a role in migraine pathophysiology. The present study aimed to determine the effect of systemic administration of NPY on TCC neuronal activity in response to dural nociceptive trigeminovascular activation. We performed in vivo electrophysiology in anesthetized rats, administered NPY (10, 30, and 100 µg·kg^−1^), and investigated the receptors involved by studying NPY Y_1_ (30 µg·kg^−1^), Y_2_ (30 µg·kg^−1^), and Y_5_ receptor agonists (100·µg·kg^−1^), and NPY Y_1_ receptor antagonist (30 µg·kg^−1^). NPY (30 and 100 µg·kg^−1^) significantly reduced TCC neuronal firing in response to dural-evoked trigeminovascular activation, but only NPY (30 µg·kg^−1^) significantly reduced spontaneous trigeminal firing. NPY Y_1_ receptor agonist also significantly reduced dural-evoked and spontaneous TCC neuronal firing. NPY (10 µg·kg^−1^), NPY Y_2_, and Y_5_ receptor agonists, and the NPY Y_1_ receptor antagonist had no significant effects on nociceptive dural-evoked neuronal firing in the TCC or spontaneous trigeminal firing. This study demonstrates that NPY dose dependently inhibits dural-evoked trigeminal activity, through NPY Y_1_ receptor activation, indicating antinociceptive actions of NPY in a migraine animal model. Based on the role of NPY in appetite regulation, it is possible that disruption of the NPY system might explain changes of appetite in migraineurs.

## Abstract 2

In vivo electrophysiology in migraine animal model shows that neuropeptide Y dose dependently inhibits dural-evoked trigeminal activity, through NPY Y1 receptor activation. Implications for pain and premonitory symptomatology.

## 1. Introduction

Migraine is considered a complex brain disorder^3,22^ and is the sixth most common cause of disability in the world.^19^ Neuroimaging studies in the premonitory phase, which represents the earliest clinical manifestations of the migraine attack,^23^ have demonstrated hypothalamic activation,^32^ while a somewhat different hypothalamic site is activated during the attack.^13^ This is in agreement with earlier animal studies,^6,7,11,31^ establishing the hypothalamus as an important brain area in migraine pathophysiology. Disturbed appetite, such as thirst, hunger, or food craving, or feeding schedules have been reported anecdotally as triggers,^9^ or as symptoms in the premonitory phase of the migraine attack.^14,18,43^ Importantly, these premonitory symptoms are regulated in part by the hypothalamus that in turn implicates appetite-related neuropeptides, including neuropeptide Y (NPY).

NPY is a peptide hormone known to be involved in pain modulation,^24^ appetite control, and circadian rhythm synchronization^30,39^ and is present in central and peripheral nervous systems, but is most abundantly expressed in the brain. In the human brain, NPY expression is highly concentrated in hypothalamic nuclei, basal ganglia, and limbic system.^1^ NPY potentially acts on six G protein–coupled receptor subtypes (Y_1-6_), with the NPY Y_3_ receptor not yet cloned. The NPY y_6_ receptor only exists as a functional receptor in the mouse and rabbit.^53^ The NPY Y_4_ receptor mRNA is the least abundant and has the most restricted distribution pattern of all NPY receptor subtypes in the central nervous system.^40^ In addition, the available agonist for the human NPY Y_4_ receptor has a short mean half-life of only approximately 6 minutes, limiting its bioactivity in vivo.^2^ NPY has been shown to be involved in antinociception in different pain models,^28,29,38^ although not studied yet in migraine. NPY causes vasoconstriction of human middle meningeal and cerebral arteries^16,25^ and inhibits plasma protein extravasation through NPY Y_2_ receptors.^52^

These data suggest that NPY could play an important role in the pathophysiology of primary headache disorders. In the present study, we used an animal model of acute dural nociceptive activation of the trigeminovascular system, which has been shown to represent a valid model to study migraine neurobiology and has reliably predicted clinical efficacy of many therapeutics, such as triptans.^5,8^ We determined the effects of intravenous administration of NPY in trigeminovascular nociceptive responses, relevant to headache, and dissected the relative contributions of specific NPY receptors. We explored NPY Y_1_, Y_2_, and Y_5_ receptor pharmacology in this animal model because mRNA encoding human NPY Y_1_ and NPY Y_2_ receptors is detected in the cerebral and meningeal arteries and in human trigeminal ganglia^47^; NPY Y_1_, Y_2_, and Y_5_ receptors are present in rodent trigeminal nucleus,^40^ and these receptors have a major role in hypothalamic appetite control.^53^ Some data have been presented previously in preliminary form.^34–36^

## 2. Materials and methods

All experiments were conducted under a license of the University of California, San Francisco Institutional Animal Care and Use Committee (IACUC) and conforming to the National Institutes of Health Guide for the Care and Use of Laboratory Animals, and adhered to the guidelines of the Committee for Research and Ethical Issues of International Association for the Study of Pain.^56^

### 2.1. Surgical preparation

Male Sprague-Dawley rats (275-365 g; n = 57, Charles River, MA) were housed (2-3 animals per cage) in temperature and light controlled rooms for at least 7 days before use with access to food and water ad libitum*.* On the day of the study at approximately 8 to 9 am, they were anesthetized with sodium pentobarbitone (60 mg·kg^−1^, intraperitoneally; Nembutal, Lundbeck, Deerfield, IL) for induction, and anesthesia was maintained with a propofol solution (PropoFlo, 25-30 mg·kg^−1^·h^−1^, intravenous infusion; Abbott Animal Health, Abbott Park, IL). During electrophysiological recording, the animals were paralyzed with pancuronium bromide (Pavulon, Hospira, Inc., Lake Forest, IL), 0.4 mg initially, and maintained with 0.2 mg every 30 minutes. The animals were monitored during the surgical procedure for changes in body temperature, respiratory rate, and blood pressure as described previously.^4^ After fixation of the skull in a stereotaxic frame (Kopf Instruments, Tujunga, CA), rats were ventilated (Ugo Basile, Comerio, Italy) with oxygen-enriched air, 2 to 2.5 ml, 80 to 100 strokes per minute, and end-tidal CO_2_ was monitored (CapStar-100; CWE Inc, Ardmore, PA) and kept between 3.5% and 4.5%. Arterial blood pressure and end-tidal CO_2_ were displayed and recorded on a personal computer using an online data analysis system (Power 1401) and Spike2 v5.21 software (Cambridge Electronic Design, Cambridge, United Kingdom). At the end of each experiment, animals were killed with a lethal dose of intravenously administered pentobarbital and phenytoin sodium (Euthasol; Virbac AH, Inc, Fort Worth, TX).

### 2.2. Middle meningeal artery and trigeminocervical complex exposure

To gain access to the dura mater and middle meningeal artery (MMA), the skull was exposed and a craniotomy of the parietal bone was performed with saline-cooled drilling and the area was covered in mineral oil. For access to the trigeminocervical complex (TCC), the muscles of the dorsal neck were separated, a cervical (C1) laminectomy was performed and the dura mater was incised to expose the brainstem at the level of the caudal medulla oblongata. A piezoelectric motor–driven microelectrode positioner attached to a micromanipulator was used to locate the optimal recording site, allowing movements of the recording electrode in 5-µm steps. After completing the surgery, the animals were left to stabilize for at least 1 hour before electrophysiological recordings.

### 2.3. Stimulation of the MMA and recording from the TCC

**Figure 1.**
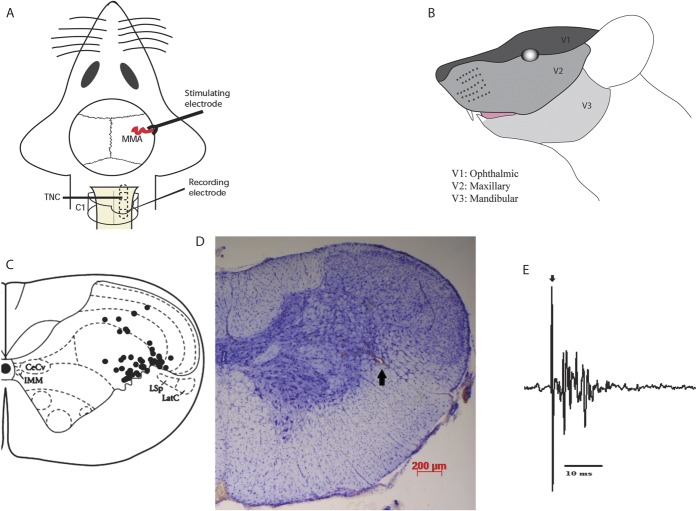
Overview of the experimental setup and neuronal characteristics. (A) Experimental setup with dural electrical stimulation and recording of neurons in the trigeminocervical complex (TCC). (B) All neurons studied were wide dynamic range, responsive to both noxious and innocuous stimulation, with cutaneous receptive field in the first (V1; ophthalmic) division of the trigeminal nerve. (C) The location of recording sites in the TCC from which recordings of nociceptive neurons, receiving convergent input from the dura mater and facial receptive field, were made. The locations were reconstructed from lesions (•) and are located in laminae III-V, predominantly in lamina V. (D) A histological example for the lesion mark (as indicated by the arrow) of the recording site in the TCC (lamina V), marked by electrothermolytic lesion (4-6 µA for 60 sec). The section was counterstained with cresyl violet. (E) An original tracing from a typical unit (second-order neurons) responding to electrical stimulation of the dura mater adjacent to the middle meningeal artery (latencies in the Aδ-fiber range). Black arrow represents stimulus artifact.

After removal of the parietal bone within the cranial window, a bipolar stimulating electrode (NE200; Rhodes Medical Instruments, Summerland, CA) connected to a stimulus isolation unit (SIU5A; Grass Instruments, Quincy, MA) was placed on the intact dura mater adjacent to the MMA for electrical stimulation of the perivascular afferents of the trigeminal nerve (Fig. 1A). Stimulation of primary trigeminal afferents was performed with supramaximal square-wave pulses generated by a Grass S88 stimulator (Grass Instruments, USA). Dural nociceptive neurons in the TCC were identified by applying square-wave electrical stimuli (8-15 V, 0.15-0.25 milliseconds, 0.4-0.5 Hz, 20 sweeps) to the dura mater. These stimulation parameters were able to activate trigeminal Aδ fibers, with approximate latencies between 3 and 20 milliseconds, and less frequently C fibers, with latencies >20 milliseconds and up to 80 milliseconds, which innervate the dura mater.

Extracellular recordings were made from neurons in the TCC, activated by dural stimulation, with cutaneous facial receptive fields (Fig. 1B), using tungsten microelectrodes (World Precision Instruments, Sarasota, FL; impedance 0.5-1.0 MΩ, measured at 1 kHz in 0.9% saline, with tip diameter of 0.5 µm). The signal from the recording electrode attached to a high-impedance headstage preamplifier (NL100AK; Neurolog, Digitimer, Welwyn Garden City, Hertfordshire, United Kingdom) was fed through an AC preamplifier (Gain x2000; Neurolog NL104). The signal was then band-pass filtered (Neurolog NL126) from 300 Hz to 20 kHz and passed through a Hum Bug 60 Hz noise eliminator (Quest Scientific, Vancouver, BC, Canada) for removal of line interference before further amplification using an AC-DC second-stage amplifier (Neurolog NL106) with a gain range of ×20 to ×90 (total gain used approximately 25,000-40,000 Hz). The obtained electrical signal was then fed through a gated amplitude discriminator (Neurolog NL201) and an analog-to-digital converter (Power 1401; Cambridge Electronic Design, Cambridge, United Kingdom) and to a microprocessor-based personal computer (Dell Latitude) using Spike 2 v5.21 software (Cambridge Electronic Design) where the signal was processed and stored. Additionally, it was fed to a loudspeaker through a power amplifier for audio monitoring (Neurolog NL120) and displayed on analog and digital storage oscilloscopes (Goldstar, LG Precision, Seoul, Korea; and Metrix Electronics, Hampshire, UK, respectively) to assist isolation of action potentials from adjacent cell activity and noise.

### 2.4. Characterization of neurons

Neurons were characterized for their cutaneous and deep receptive fields. The cutaneous receptive field was assessed in all 3 territories of the trigeminal innervation and identified as the recording electrode was advanced in the spinal cord. The receptive field was assessed for both nonnoxious inputs, with gentle brushing using a cotton tip applicator, and noxious inputs, with pinching with forceps that was painful when applied to humans. When a neuron sensitive to the stimulation of the ophthalmic (V1) dermatome of the trigeminal nerve was identified, it was tested for convergent input from the dura mater.

### 2.5. Experimental design

Trains of 20 stimuli were delivered at 5-minute intervals to assess the baseline response to dural electrical stimulation. Responses were analyzed using poststimulus histograms with a sweep length of 100 milliseconds and a bin width of 1 milliseconds that separated Aδ-fiber-activated (3-20 milliseconds) and C-fiber-activated (20-80 milliseconds) firing. Spontaneous activity (spikes per second, Hz) was recorded for 150 s preceding the dural stimulation using peristimulus histograms. Background activity was analyzed as cumulative rate histograms in which neuronal activity gated through the amplitude discriminator was collected into successive bins.

When stable baseline values of the stimulus-evoked responses were achieved (mean of 3 stimulation series that would not differ more than 10%) and cutaneous and deep receptive field inputs from the ophthalmic division of the trigeminal nerve were obtained, responses were tested for up to 60 minutes after pharmacological or vehicle intervention.

### 2.6. Drugs

TCC neuronal responses to electrical dural afferent stimulation were tested with NPY human (Sigma-Aldrich) in a dosage of 10 µg·kg^−1^ (n = 6), 30 µg·kg^−1^ (n = 9), and 100 µg·kg^−1^ (n = 8). The dose of NPY at 10 µg·kg^−1^ (intravenously) was chosen based on studies of experimental vasodilation in the dura mater^49^ and escalated thereafter for a dose–response effect.

To investigate NPY receptor pharmacology, 3 different NPY receptor agonists (Tocris Bioscience, Minneapolis, MN) were administered intravenously: NPY Y_1_ receptor agonist ([Leu31,Pro34]-NPY [human], 30 µg·kg^−1^, n = 10); NPY Y_2_ receptor agonist (PYY 3-36, 30 µg·kg^−1^, n = 6); and NPY Y_5_ receptor agonist ([cPP1-7,NPY19-23,Ala31,Aib32,Gln34]—hPancreatic Polypeptide, 100 µg·kg^−1^, n = 6). The doses of the agonists were selected on the basis of earlier studies in which NPY exhibited subnanomolar affinities to the Y_1_, Y_2_, and Y_5_ receptors and nanomolar binding affinity to the Y_4_ receptor.^12^ In addition, each of the Y_1_, Y_2_, and the Y_5_ receptor agonists used in this study show similar binding affinities to their receptors, 0.39 nM,^37^ 0.3 nM,^15^ and 0.24 nM,^12^ respectively. Thus, based on the binding of NPY to each receptor, and the similarity of responses to each agonist, similar doses were chosen. Finally, to determine whether there was any endogenous tonic effects of the NPY Y_1_ receptor in the modulation of trigeminal nociceptive transmission, a highly selective NPY Y_1_ receptor antagonist (BVD 10) in a dosage of 30 µg·kg^−1^ (n = 5) was administered alone using the same scheme described above. The optimal dose of the antagonist was derived from preliminary experiments showing 30 µg·kg^−1^ produced a maximum blood pressure increase of 11 ± 1%. Sterile water alone was used as the vehicle control group (n = 7) and naratriptan in a dosage of 10 mg·kg^−1^ (n = 6) as a positive control to these responses. All drugs were dissolved in sterile water.

### 2.7. Postsurgical examination of tissue

At the end of the experiment and after terminal anesthesia, an electrothermolytic lesion was made in the TCC by passing a current down the recording electrode (4-6 µA for 60 s) to confirm the location of the recording electrode. The brain and cervical spinal cord were removed. Serial 60-µm-thick coronal sections were cut from the medulla oblongata and C1-C3 spinal segments, stained for cresyl violet, and visualized under the light microscope (Axioplan Microscope; Carl Zeiss GmbH, Jena, Germany), using the rat brain atlas Paxinos and Watson, 2005^41^ for reference.

### 2.8. Statistical analysis

Data collected for Aδ fibers represent the normalized data for the number of cells firing over a 10-milliseconds time period in the region 4 to 20 milliseconds poststimulation over the 20 collections and expressed as mean ± SEM. Spontaneous activity was measured in cell firings per second (Hz). Statistical analysis was performed using IBM SPSS 20.0 software. To measure whether there was a significant effect across the 60-minutes time course within a drug group of animals, we used analysis of variance for repeated measures with Bonferroni post hoc correction for multiple comparisons. If Mauchly test of sphericity was violated, appropriate corrections to degrees of freedom were made according to Greenhouse–Geisser.^17^ Student paired *t* test was used for post hoc analysis of the significance of individual time points, using the average of the 3 baselines for comparison. Statistical significance was assumed at *P* < 0.05.

## 3. Results

### 3.1. Electrophysiological data

Recordings were made from a total of 63 neurons in 57 rats. Animals studied had a mean body weight of 319 ± 3.2 g. Extracellular recordings in the TCC were made from wide-dynamic-range neurons, responsive to dural (MMA) stimulation (Fig. 1A), and with cutaneous receptive fields in the ophthalmic division (V1) of the trigeminal nerve (Fig. 1B). Most neurons were located in lamina V of the dorsal horn at the level of the cervicomedullary junction (with a few neurons in laminae III-IV), at an average depth of 476 ± 27 µm, and the electrode placement was confirmed in all animals by an electrothermolytic lesion in the TCC (Fig. 1C, D). Neurons responding to dural electrical stimulation responded with an average latency of 10.8 ± 0.2 milliseconds (range 4-20 milliseconds, an example of evoked neuronal firing can be seen in Fig. 1E) and hence were classified as Aδ fibers. The mean ongoing spontaneous firing rate was 23.8 ± 2.0 Hz with most neurons responding between 10 and 20 Hz; this is within the same range as that demonstrated in previous studies.^4^

### 3.2. Controls

Intravenous injection of sterile water (vehicle control) had no significant effect on Aδ-fiber responses (F_3.1,18.8_ = 0.9; *P* = 0.42) and ongoing spontaneous activity (F_1.8,10.9_ = 0.3; *P* = 0.72) of trigeminal second-order neurons during the 60-minutes time period.

Naratriptan 10 mg·kg^−1^ (Sigma-Aldrich) was also tested in another group of animals as a positive control for inhibition of second-order neurons.^21^ Extracellular recordings showed a significant decrease of dural-evoked nociceptive responses (F_2.2,10.9_ = 3.9; *P* = 0.048) throughout the 60 minutes, reaching a maximum inhibition of 27% at 30 minutes (*t*_5_ = 3.592; *P* = 0.016) and 60 minutes (*t*_5_ = 4.663; *P* = 0.006). Ongoing spontaneous background activity was also significantly reduced (F_2.1,10.5_ = 12.4; *P* = 0.002) within the TCC after naratriptan administration across the 60-minutes cohort. A maximum inhibition of 58% was achieved at 60 minutes when compared with the baseline (*t*_5_ = 4.472; *P* = 0.007).

### 3.3. NPY dose dependently inhibits neurotransmission in the trigeminovascular system

NPY (10 µg·kg^−1^) did not have any significant effect on dural-evoked nociceptive firing in the TCC (F_2.2,11.1_ = 0.3; *P* = 0.78) or the ongoing spontaneous activity (F_1.6,8.2_ = 1.3; *P* = 0.30). NPY (30 µg·kg^−1^) significantly inhibited dural-evoked responses (F_8,64_ = 3.8; *P* = 0.001), with a maximal inhibition of 17% at 60 minutes (*t*_8_ = 3.284; *P* = 0.011). Ongoing spontaneous activity was also significantly inhibited (F_8,64_ = 2.7; *P* = 0.012) over the 60-minutes cohort, with a maximum inhibition of 23% at 60 minutes when compared with baseline (t_8_ = 2.507; P = 0.037). Likewise, NPY (100 µg·kg^−1^) significantly reduced nociceptive neuronal firing within the TCC (F_3.1,21.5_ = 8.5; *P* = 0.001). Maximal inhibition of 20% was achieved at 30 minutes when compared with baseline (*t*_7_ = 5.645; *P* = 0.001). At this dose, there was no significant effect on spontaneous neuronal firing (F_3.6,25.2_ = 0.6; *P* = 0.67). The dural-evoked neuronal responses of human NPY (30 µg·kg^-1^ and 100 µg·kg^−1^) did not return to baseline 60 minutes or 90 minutes after the administration (data not shown).

### 3.4. NPY Y_1_ receptor agonist significantly reduces the activation of the trigeminovascular system

To dissect the specific NPY receptor that mediates this inhibitory response in the trigeminovascular system, we also administered selective agonists. The NPY Y_1_ receptor agonist (30 µg·kg^−1^) significantly reduced the dural-evoked neuronal firing (F_8,72_ = 3.9; *P* = 0.001), reaching a maximum inhibition of approximately 22% at 15 minutes (*t*_9_ = 4.419; *P* = 0.002). It also significantly reduced the ongoing spontaneous responses (F_8,72_ = 2.4; *P* = 0.022), with the greatest point of inhibition at 45 minutes, by 29% below baseline firing (*t*_9_ = 2.545; *P* = 0.031). Administration of the NPY Y_2_ receptor agonist (30 µg·kg^−1^) had no significant effect on nociceptive dural-evoked firing (F_1.6,8.1_ = 0.3; *P* = 0.70) or ongoing spontaneous neuronal firing (F_1.9,9.9_ = 1.0; *P* = 0.386).

In a pilot study using the NPY Y_5_ receptor agonist (30 µg·kg^−1^), there was no significant difference in neuronal firing compared with baseline values (data not shown); thus, we decided to perform the next experiments using a dose of 100 µg·kg^−1^. NPY Y_5_ receptor agonist (100 µg·kg^−1^) had no significant effect on dural-evoked nociceptive responses (F_3.1,15.7_ = 0.8; *P* = 0.51) and ongoing spontaneous neuronal activity (F_1.4,6.8_ = 1.0; *P* = 0.375) in the TCC.

**Figure 2.**
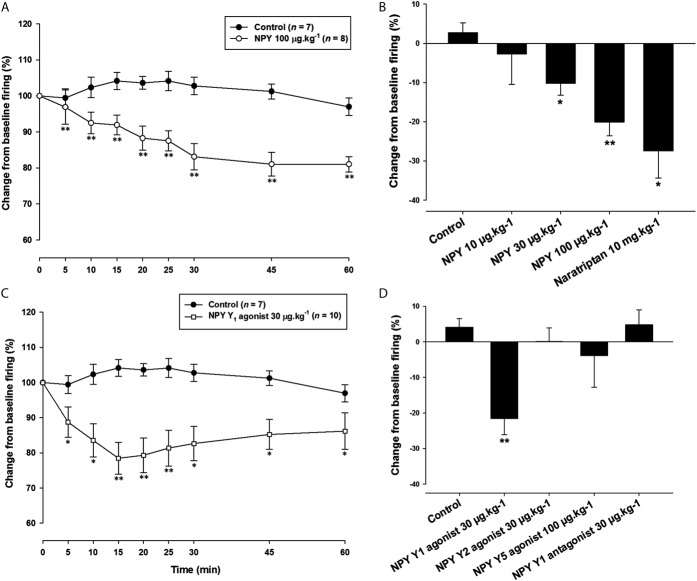
Effects of neuropeptide Y (NPY) and selective NPY Y receptor modulation on dural-evoked neuronal firing in the trigeminocervical complex (TCC). (A) Time course changes in the average response of dural-evoked Aδ-fiber trigeminal neuronal firing in response to human NPY (100 µg·kg^−1^), which significantly decreased neuronal responses across the 60-min study. Vehicle has no effect on responses. (B) Bar graph of the maximum effect at the “30-minutes time point” of the change from baseline of dural-evoked Aδ-fiber activity in the TCC. NPY (10-100 µg·kg^−1^) dose dependently inhibited dural-evoked neuronal responses, with both 30 and 100 µg·kg^−1^ significantly inhibiting dural-evoked Aδ-fiber activity in the TCC. Water for injection (control) had no effect and naratriptan (10 mg·kg^−1^) also significantly inhibited responses. (C) Time course changes in the average response of dural-evoked Aδ-fiber trigeminal neuronal firing in response to a selective NPY Y_1_ agonist (30 µg·kg^−1^), which significantly decreased trigeminal responses across the 60-minutes study. Vehicle has no effect on responses. (D) Bar graph of the maximum effect at the “15-minutes time point” of the change from baseline of dural-evoked Aδ-fiber activity in the TCC after selective NPY receptor agonists and Y_1_ antagonist. Only the NPY Y_1_ receptor agonist (30 µg·kg^−1^) significantly inhibited dural-evoked Aδ-fiber activity in the TCC. The selective NPY Y_2_ and Y_5_, and Y_1_ antagonist had no effect on responses. Data are presented as mean ± SEM; *P< 0.05, **P < 0.005 significance when compared with an average of the 3 baselines using Student paired *t* test.

Taken together, these data demonstrate that NPY is able to inhibit the effects of dural-evoked trigeminovascular activation in a dose-dependent manner (Fig. 2A, B) and these effects seem to be specific to the NPY Y_1_ receptor (Fig. 2C, D).

### 3.5. NPY Y_1_ receptor antagonist has no significant effect on the activation of the trigeminovascular system

Intravenous administration of a highly selective NPY Y_1_ receptor antagonist (30 µg·kg^−1^) had no significant effect on Aδ-fiber responses to dural stimulation (F_3.0,12.0_ = 0.8; *P* = 0.51) or ongoing spontaneous neuronal firing (F_1.4,5.6_ = 0.8; *P* = 0.45).

### 3.6. Blood pressure effect

**Figure 3.**
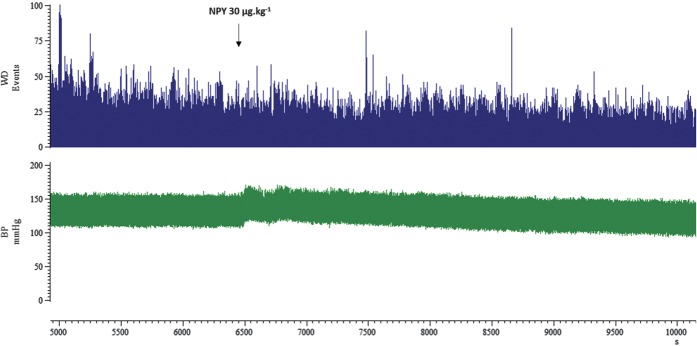
Blood pressure (BP) effects of neuropeptide Y (NPY). Original recording of BP and spontaneous trigeminocervical complex (TCC) neuronal firing before intravenous injection of NPY 30 µg·kg^−1^ and for the following 60 minutes. The BP increased (8%) following injection and slowly returned to baseline levels within 15 minutes; it was reduced by 8% at 60 minutes compared with baseline. In contrast, maximal spontaneous TCC neuronal inhibition occurred at 60 minutes, here to 40% of the baseline.

In all experiments, blood pressure was at physiological levels (92.6 ± 2.4 mm Hg) before injections. Intravenous administration of NPY human 10, 30, and 100 µg·kg^−1^ significantly increased mean arterial blood pressure immediately after injection. NPY human 10, 30, and 100 µg·kg^−1^ reached a maximum increase of 16 ± 5% (*t*_5_ = 3.954; *P* = 0.011), 17 ± 2% (*t*_8_ = 6.334; *P* = 0.000), and 49 ± 5% (*t*_7_ = 7.241; *P* = 0.000), respectively. Blood pressure values slowly recovered to preinjection values after approximately 12 ± 3 minutes (*t*_5_ = 3.296; *P* = 0.022), 10 ± 2 minutes (*t*_8_ = 5.525; *P* = 0.001), and 19 ± 3 minutes (*t*_7_ = 6.500; *P* = 0.000) postinjection, respectively. Original data showing blood pressure and spontaneous TCC neuronal firing before intravenous injection of NPY 30 µg·kg^−1^ and for the following 60 minutes can be seen in Fig. 3. Blood pressure slowly returned to baseline levels within 15 minutes independent of the maximal dural-evoked and spontaneous TCC neuronal inhibition, which occurred at 60 minutes postinjection.

NPY Y_1_ receptor agonist (30 µg·kg^−1^) significantly increased the mean arterial blood pressure, maximally by 67 ± 5% (*t*_9_ = 17.720; *P* = 0.000), slowly returning to baseline after 16 ± 5 minutes (*t*_9_ = 3.149; *P* = 0.012). However, the NPY Y_2_ and the NPY Y_5_ receptor agonist had no significant effects on blood pressure. The NPY Y_1_ receptor antagonist (30 µg·kg^−1^) reached a maximum increase of 11 ± 1% (*t*_4_ = 8.948; *P* = 0.001) after injection, returning to preinjection values after 14 ± 2 minutes (*t*_4_ = 4.740; *P* = 0.009). Naratriptan decreased the blood pressure by 8 ± 5% (*t*_5_ = 1.49; *P* = 0.195) after injection and never returned to preinjected blood pressure values.

## 4. Discussion

The data demonstrate that NPY can modulate nociceptive trigeminovascular transmission in second-order neurons of the TTC after peripheral systemic administration. This effect could be mimicked by an NPY Y_1_ receptor agonist but not by NPY Y_2_, and Y_5_ receptor agonists. Moreover, there was no resting effect of the NPY Y_1_ receptor on ongoing spontaneous trigeminal firing as evidenced by no effect of an NPY Y_1_ receptor antagonist, and consistent with the modest response and lack of a dose–effect for NPY itself. These data are consistent with the existence of an inhibitory NPY Y_1_ receptor in the TTC. These results are in agreement with the antinociceptive role of NPY in other animal models of different pain states.

Our findings reveal a potential involvement of NPY in migraine pathophysiology, through its ability to modulate the firing of dural-evoked nociceptive second-order trigeminovascular neurons. These data contrast with previous studies that also used dural nociceptive stimulation. In these previous experiments, neuropeptide levels were measured in the extracranial vasculature, taken from external jugular vein blood, after superior sagittal sinus stimulation and were unchanged,^55^ in contrast to calcitonin gene-related peptide^55^ and pituitary adenylate cyclase–activating peptide.^54^ However, this may reflect the locus of release and action in the central nervous system in contrast to the more widespread release of other neuropeptides involved in migraine. For example, localized release of NPY within hypothalamic nuclei, where we know it is released during feeding and impact migraine symptoms, would not be detected in such assays. It is also important to reflect that NPY readily gets into the brain, and our data demonstrate that it is able to attenuate the responses of second-order trigeminovascular neurons to dural-evoked stimulation.

A further aim was to investigate whether NPY exerts tonic antinociceptive control of TCC neurons, by administering an NPY Y_1_ receptor antagonist in our model. Spinal NPY is known to exert a tonic, long-lasting inhibitory control of spinal nociceptive transmission, as demonstrated by NPY knockdown or intrathecal administration studies.^45^ We did not see an effect on neuronal firing after NPY Y_1_ antagonist administration, which suggests that NPY does not exert a tonic control of TCC neuronal firing.

Concerning the cardiovascular effects in our study, the inhibitory response of NPY is not due to blood pressure changes since the time course of blood pressure elevation is different from the inhibition of the trigeminal neurons within the TCC. This is confirmed when the blood pressure returns to baseline values at some point of the study and the TCC neuronal firing is significantly inhibited across the 60-min study.

In our study, we chose to administer NPY systemically. This allowed us to directly translate findings into the clinical setting for potential therapeutics, and because we know NPY is able to cross the blood–brain barrier,^27^ it suggests it is likely to acting centrally. Based on this probable action in the brain, we believe the most likely targets in modulating trigeminovascular nociceptive transmission in this study are at the level of the hypothalamus and also directly on the TCC neurons. We have tried to rationalize these potential sites of action with what we know of NPY and how it might fit into a role in migraine pathophysiology. Reported migraine triggers (skipping meals) or symptoms (appetite changes) might act through NPY because altered feeding behavior is able to interfere with NPY pathways. For example, NPY mRNA expression is upregulated by fasting in the hypothalamic arcuate nucleus (ARC)^48^; NPY Y_1_ receptor mRNA is elevated in the hypothalamus in response to fasting and food restriction^50^; and following food deprivation, NPY is released from ARC-projecting neurons into the hypothalamic paraventricular nucleus (PVN) in vivo.^26^ The PVN is a pivotal nucleus with second-order neurons that integrates signals from ARC first-order neurons (such as NPY-containing neurons), and then sends downstream information to the nucleus tractus solitarii (NTS) to control appetite.^10^ Specific hypothalamic nuclei are thought to have a major role in migraine pathophysiology.^33^ It has been shown that PVN modulates basal and nociceptive meningeal-evoked activity of trigeminal neurons, containing neurons known to project directly to the trigeminal nucleus caudalis.^42,46^ Taking into consideration that NPY activity within the ARC-PVN neural circuit is seen as a homeostatic response to perturbations of energy balance, our results showing an inhibition of dural-evoked nociception and spontaneous TCC neuronal firing might indicate that NPY could be acting within the PVN to modulate the TCC through direct projections.

In addition to this, ARC neurons project to many pain modulatory nuclei in the brainstem, including the periaqueductal gray, nucleus tractus solitarii, dorsal raphe nucleus, and locus coeruleus,^44^ with NPY-immunoreactive neuronal projections from the ARC to dorsal raphe nucleus and locus coeruleus in the rat.^51^ Moreover, intra-ARC administration of NPY exerts an antinociceptive effect in intact rats and in rats with inflammation through NPY Y_1_ receptor activation.^29^ Because ARC contains a high concentration of NPY-producing neurons and NPY receptors, systemic NPY in our study could also act directly in the ARC with further actions either in the PVN or other pain modulation nuclei to induce inhibition of nociceptive activation of trigeminovascular neurons.

In conclusion, hypothalamic nuclei are in key positions to mediate the triggering of migraine and trigeminovascular activation, modulating migrainous symptoms through appetite changes. In this study, we show that NPY, through its NPY Y_1_ receptor, may be involved in such a pathway. The data offer further pharmacologic therapeutic targets to those currently under development,^20^ although there is a chance of increased appetite with the NPY Y_1_ receptor agonist approach, which could be offset by chronic systemic administration of low doses of an NPY Y_1_ agonist. Investigating the pathophysiological mechanisms taking place during the earlier phase of the migraine attack is clearly important to understand migraine and its associated symptoms.

## Conflict of interest statement

P. J. Goadsby reports, unrelated to this report, grants, and personal fees from Allergan, grants and personal fees from eNeura Inc, personal fees from Autonomic Technologies Inc, grants and personal fees from Amgen Inc, personal fees from Alder Biopharmaceuticals, personal fees from Pfizer Inc, personal fees from Dr Reddy's Laboratories, personal fees from Zosano Pharma Corporation, personal fees from Colucid Pharmaceuticals, Ltd, personal fees from Eli-Lilly and Company, personal fees from Avanir Pharmaceuticals, personal fees from WL Gore and Associates, personal fees from Heptares Therapeutics, personal fees from Nupathe Inc, personal fees from Teva, personal fees from Cipla Ltd, personal fees from Ajinomoto Pharmaceuticals Co, personal fees from Akita Biomedical, personal fees from Wells Fargo, personal fees from Ethicon, US, personal fees from EMKinetics, personal fees from Promius Pharma, personal fees from Supernus, personal fees and other from Trigemina, personal fees from MedicoLegal work, personal fees from Journal Watch, personal fees from Up-to-Date, outside the submitted work; In addition, Dr. Goadsby has a patent Magnetic stimulation for headache pending. The other authors have no conflicts of interest to declare.

M. Martins-Oliveira is grateful to the Portuguese Fundação para a Ciência e Tecnologia (FCT) for its support with an individual PhD grant (SFRH/BD/77127/2011). The work has been funded by EUROHEADPAIN European Union FP7 and the Wellcome Trust.

Author contributions: All the authors have read and approved the manuscript. M. Martins-Oliveira conducted and designed experiments, performed data analyses and data interpretation and wrote the manuscript. S. Akerman assisted in in vivo electrophysiology experiments, designed experiments, contributed to interpretation of data and revised critically the manuscript. I. Tavares contributed to interpretation of data and revised critically the manuscript. P. J. Goadsby designed experiments, contributed to interpretation of data and revised critically the manuscript.
